# Lattice distortion induced internal electric field in TiO_2_ photoelectrode for efficient charge separation and transfer

**DOI:** 10.1038/s41467-020-15993-4

**Published:** 2020-05-01

**Authors:** Yuxiang Hu, Yuanyuan Pan, Zhiliang Wang, Tongen Lin, Yuying Gao, Bin Luo, Han Hu, Fengtao Fan, Gang Liu, Lianzhou Wang

**Affiliations:** 1 Nanomaterials Centre, Australian Institute for Bioengineering and Nanotechnology, and School of Chemical Engineering, The University of Queensland, Brisbane, QLD 4072 Australia; 2 State Key Laboratory of Heavy Oil Processing, College of Chemical Engineering, China University of Petroleum (East China), Qingdao, 266580 China; 3 State Key Laboratory of Catalysis, Dalian National Laboratory for Clean Energy, The Collaborative Innovation Centre of Chemistry for Energy Materials (iChEM), Dalian Institute of Chemical Physics, Chinese Academy of Sciences, Dalian, 116023 China; 4 Shenyang National Laboratory for Materials Science, Institute of Metal Research, Chinese Academy of Sciences, 72 Wenhua Road, Shenyang, 110016 China; 5 School of Materials Science and Engineering, University of Science and Technology of China, 72 Wenhua Road, Shenyang, 110016 China

**Keywords:** Electrochemistry, Solid-state chemistry, Photocatalysis, Electronic properties and materials

## Abstract

Providing sufficient driving force for charge separation and transfer (CST) is a critical issue in photoelectrochemical (PEC) energy conversion. Normally, the driving force is derived mainly from band bending at the photoelectrode/electrolyte interface but negligible in the bulk. To boost the bulky driving force, we report a rational strategy to create effective electric field via controllable lattice distortion in the bulk of a semiconductor film. This concept is verified by the lithiation of a classic TiO_2_ (Li-TiO_2_) photoelectrode, which leads to significant distortion of the TiO_6_ unit cells in the bulk with well-aligned dipole moment. A remarkable internal built-in electric field of ~2.1 × 10^2^ V m^−1^ throughout the Li-TiO_2_ film is created to provide strong driving force for bulky CST. The photoelectrode demonstrates an over 750% improvement of photocurrent density and 100 mV negative shift of onset potential upon the lithiation compared to that of pristine TiO_2_ film.

## Abstract (web summary)

The driving force for charge transfer in photoelectrochemical systems is typically derived from band bending at a surface-electrolyte interface. In this work, battery-type lithiation of TiO_2_ generates a built-in electric field in the bulk material, giving a 750% enhancement in photocurrent density.

## Introduction

Photoelectrochemical (PEC) water splitting provides an attractive approach to utilize abundant solar energy for renewable hydrogen production^1^. The PEC performance heavily relies on three sequential steps, namely light absorption, charge separation and transfer (CST) of photogenerated charges, and their surface reactions on the semiconductor photoelectrodes^1–3^. Among these steps, the CST process is normally driven by the electric field associated with the band bending in surface space charge layer of the semiconductor photoelectrodes exposed to the electrolytes^4–6^. However, a limited width of such a space charge layer (*W*_SC_ ~ 100 nm) cannot sufficiently guarantee the charge separation in the whole film photoelectrodes usually with the thickness of several hundreds of nanometers up to micrometers. For this reason, serious charge recombination normally occurs in the CST process, because there is no effective electric field in the majority of bulky photoelectrodes, apart from very weak carrier concentration gradient. Accordingly, the low CST efficiency is one of the most challenging problems in the PEC water splitting process^3,6–10^. How to create an additional strong driving force in the bulk of the photoelectrodes to improve the CST has been an important research question to address.

Some efforts have been devoted to extending the width of built-in electric field, such as gradient doping^11,12^. Nevertheless, most of the resultant structures are actually analogous to multi-junction structures, which create different space charge regions at the interfaces but do not create a desirable bulk band bending^6^. Creating an effective electric field throughout the whole photoelectrode for the promoted CST process remains highly challenging. We were interested in solving this problem by revisiting the basic crystal structure of the semiconductor materials. In principle, if the symmetry of a crystal structure was deliberately broken, a dipole moment can be expected because of the displacement of positive and negative charge centers of the unit cells^13^. In this scenario, a significant electric field will be built up along the dipole direction throughout the whole semiconductor film, as a result of which the bulk band bending (i.e., the driving force of CST) will be possible^14^. An alternative way to create dipoles is to induce Jahn-Teller effect by lattice distortion (such as tension/compression of the unit cell)^15,16^. Although the developed strategies such as doping can create local lattice distortions, the randomly distributed dipoles in the bulk will cancel out each other, leading to a negligible built-in electric field^15^. Thus, innovative designs that can precisely tune the lattice distortion in the bulk of photoelectrodes for the generation of controllable dipole moment are urgently needed for creating effective bulky driving force to accelerate the PEC process.

Herein, we report a rational approach to design built-in electric field in the bulk of a prototypical TiO_2_ photoelectrode by using a lithiation process in the lithium ion battery (LIB) discharge process. Our idea was inspired by the lithiation/de-lithiation process for precisely tailoring the structure and lattice strain in electrocatalysts^20–23^. The lattice distortion and dipole moment generated upon the controllable Li-ion intercalation in TiO_2_ (Li-TiO_2_) was predicted by density functional theory (DFT) simulation and further experimentally confirmed by characterizations of bulk Li-TiO_2_ structure. The electric field stemmed from dipole moment in the bulk was evidenced by Kelvin probe force microscope (KPFM). The average electric field strength throughout the Li-TiO_2_ film was measured as high as 2.1 × 10^2^ V m^−1^, compared with the negligible electric field measured in pristine TiO_2_ film. As a result, the photogenerated CST efficiency of Li-TiO_2_ photoelectrode was drastically stimulated with an over 7.5 times improvement in photocurrent and an onset potential reduction by 100 mV compared with that in pristine TiO_2_. This result highlights the significant role of bulk electric field in the PEC process.

## Results

### Mechanism of lithium electrochemically tuned TiO2 photoanode

**Fig. 1:**
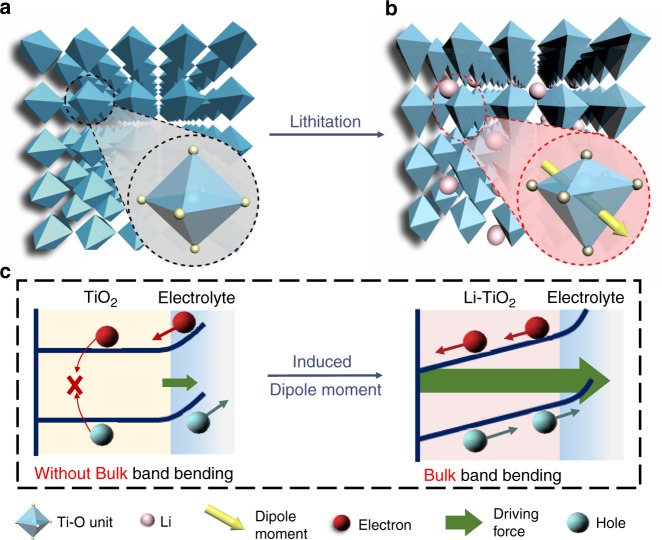
Schematic changes of anatase TiO2 upon lithiation. The schematic bulk lattice structures of (**a**, **b**) pristine and lithiated TiO_2_. Insets in **a** and **b** are the typical TiO_6_ unit cells without and with the distortion, which leads to asymmetric Ti-O bonds and generates a dipole moment as presented by the arrow in **b**. **c** Schematic of the band bending and driving force in the TiO_2_ and Li-TiO_2_ bulks without and with the lattice distortions induced dipole moments, respectively. The band bending and driving force exists only in surface layer of TiO_2_ but in both surface layer and bulk of Li-TiO_2_.

TiO_2_ is not only a most studied semiconductor in PEC research, but also an insertion-type anode material in LIBs^17,18^. This allows us to intentionally tune the crystal structure of TiO_2_ borrowing the lithiation strategy in LIBs. One important feature of lithiation process is that the degree of lattice distortion can be finely tailored by precisely controlling lithium content at different stages of discharge^19–22^. When Li ions are inserted, the TiO_2_ (anatase phase) crystal structure will be uniformly distorted in three dimensions (i.e., Jahn-Teller effect) to accommodate the external Li cations (Fig. 1a, b). This process leads to the distortion of TiO_6_ units in the TiO_2_ crystal (inset of Fig. 1a, b). The TiO_6_ units in pristine TiO_2_ are octahedra with high symmetry and have the negative charge centers of anions (O atoms) overlapping with the positive charge centers of cations (Ti atom). In strong contrast, after lithiation, the significant Jahn-Teller effect leads to the mismatch between the positive and negative charge centers. As a result, the dipoles are generated in the TiO_6_ units and dipole moment can be calculated accordingly (inset of Fig. 1b)^23^. More interestingly, when all the well-aligned TiO_6_ units in the TiO_2_ crystal have the distortion with the same directed dipoles, an additional electric field is created along with the direction of dipole moment throughout the entire TiO_2_ film. Thus, a bulky band bending can be expected in the whole TiO_2_ photoelectrode as illustrated in Fig. 1c^24^.

### Simulation of lithium electrochemically tuned TiO_2_ photoanode based on DFT

**Fig. 2:**
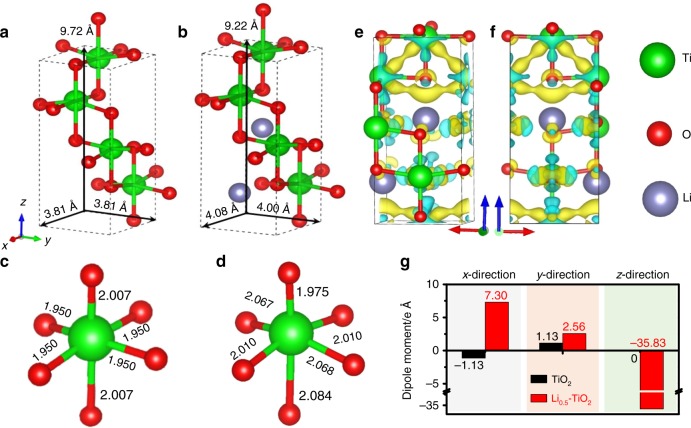
Theoretical simulation of lattice distortion and dipole moment. **a**, **b** The lattice parameters of the structurally optimized anatase TiO_2_ and orthorhombic Li_0.5_TiO_2_ cells. **c**, **d** The theoretically determined Ti-O bond lengths of the unit cells of TiO_2_ and Li_0.5_TiO_2_. **e**, **f** Deformation charge densities of Li_0.5_TiO_2_ from two different lateral views of the *x*–*z* plane. The light yellow and blue surfaces represent the charge gain and loss, respectively. **g** The calculated dipole moments of TiO_2_ and Li_0.5_TiO_2_ along three different crystallographic directions.

To verify our hypothesis, we first carried out the first-principle simulation to investigate the influence of Li-ion intercalation. Tetragonal structure of intrinsic anatase TiO_2_ (Fig. 2a) is highly symmetric with the lattice parameters of *a* = *b* = 3.81 Å and *c* = 9.72 Å. When the Li ions are inserted into the lattices (the Li_0.5_TiO_2_ is selected as the representative model), the crystal structure changes from tetrahedral to orthorhombic with the lattice parameters of *a* = 4.08 Å, *b* = 4.00 Å, and *c* = 9.22 Å as exhibited in Fig. 2b. This process requires a formation energy of 1.34 eV (see Supplementary Information for detailed calculation). These results indicate that the unit cell of TiO_2_ is totally distorted in all three dimensions upon Li-ion insertion accompanying the high energy input. In Fig. 2c and d, a close insight into the TiO_6_ octahedra gives that the length of two symmetric Ti-O bonds along the *z*-axial direction ([001] direction of the TiO_2_ crystal) significantly changes from 2.007 Å to 1.975 and 2.084 Å of two asymmetric bonds upon Li-ion insertion, while the length of four equivalent Ti-O bonds along *x*, *y*-direction is also slightly stretched. The changes of Ti-O bond length have been evidenced in previous research via X-ray absorption studies where the Ti-O bond lengths were split into two sets during the lithiation^25^.

According to the charge density distribution of TiO_2_ and Li_0.5_TiO_2_ (Supplementary Fig. 1 and Supplementary Table 1), the Bader charge of individual atoms in the crystal cell of Li_0.5_TiO_2_ was also calculated and summarized in Supplementary Table 1. Each Li atom contributes 0.85 electron as donor. Moreover, the deformation charge density (Δ*ρ*) in Fig. 2e and f from two lateral views of *x*–*z* plane clearly shows the asymmetric distribution of charge density, where the negative charge centers (gaining electrons) are deviated from the positive charge centers (losing electrons). Further analysis of the dipole moment of TiO_2_ and Li_0.5_TiO_2_ in Fig. 2g suggests that the dipole moment does not change much along the *x*- and *y*-direction but drastically increases to −35.83 e Å along the *x*- and *y*-directions in the lithiated TiO_2_ (Li_0.5_TiO_2_), indicating a strong built-in electric field along the [001] direction of the TiO_2_ crystal.

### Material characterization of lithiated TiO_2_

**Fig. 3:**
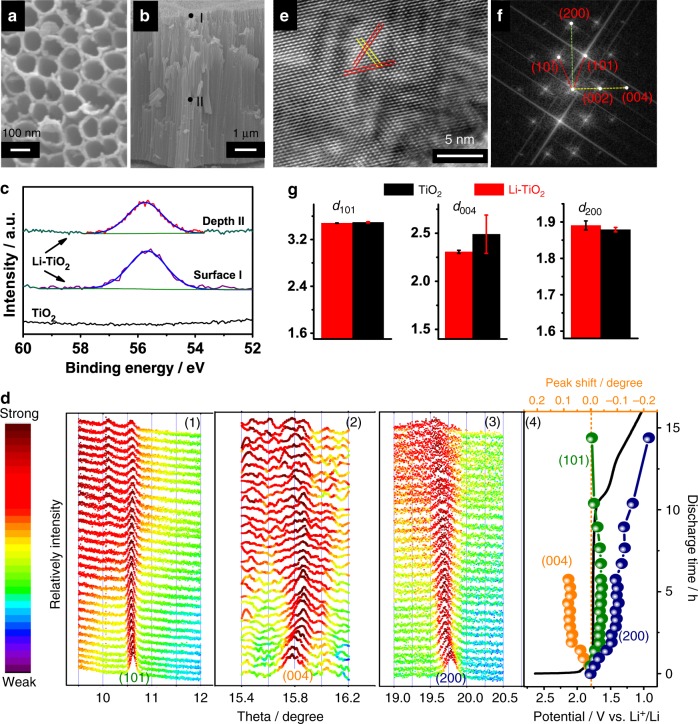
The structure change of TiO2 upon the lithiation. **a**, **b** The top-view and cross-section view SEM images of Li-TiO_2_ electrode. **c** XPS spectra of Li 1 s recorded from the top surface of TiO_2_ and Li-TiO_2_. The signal from the exposed bulk (marked point II in **b**) of Li-TiO_2_ was also recorded. **d** Evolution of in-situ XRD patterns of TiO_2_ at the discharging process in LIBs (black curve in (4)). Three peaks (1) (101), (2) (004), and (3) (200) are traced. The peak shifts referred to the ones at initial peaks are summarized in (4). **e**, **f** A typical HR-TEM image of Li-TiO_2_ and its corresponding FFT image. **g** Comparison of the lattice distances along [101], [004], and [200] for (black) TiO_2_ and (red) Li-TiO_2_.

To verify the proposed hypothesis and simulation results, it is of primary importance to confirm the lattice distortion during Li-ion insertion. Anatase TiO_2_ tubes aligned on Ti substrate prepared by the anodization of Ti foil were first used for the lithiation^26^. The lithiation process does not change the tube morphology (Supplementary Fig. 2). The scanning electron microscopy (SEM) images in Fig. 3a and b show that the tubes have a diameter of ~80 nm (Fig. 3a), wall thickness of ~20 nm (Supplementary Fig. 2), and length of ~7 μm (Fig. 3b). The large diameter and wall thickness make these tubes significantly different from the TiO_2_ nanotubes with ultrathin wall of ~1 nm and very small diameter of ~10 nm prepared by hydrothermal routes^27–29^; thus, the structure of these tubes can be considered to be close to bulky TiO_2_. On the other hand, both the large tube diameter and strong interfacial contact between the tubes and highly conductive Ti substrate^30,31^ facilitate the Li-ion diffusion into the whole tube walls during the discharge process to form a uniform lithiation, which is vital to the generation of lattice distortion and related dipole effect anticipated. The discharge duration was controlled to tune the Li content in the Li-TiO_2_ (Supplementary Fig. 3). The optimized Li-TiO_2_ photoelectrode was obtained by discharging for around 5 h with Li content close to the theoretical model (Li_0.5_TiO_2_). Comparison of the X-ray photoelectron spectroscopy (XPS) spectra of Li 1 s peak (Fig. 3c) recorded from the different parts of the pristine and lithiated tubes confirms the effective insertion of Li ions in the Li-ion inserted TiO_2_ (Li-TiO_2_). Moreover, the very similar signal intensity of Li in both the top surface (marked point **I** in Fig. 3b) and bulk (marked point **II** in Fig. 3b after removing the top active material by argon ion sputtering) of the Li-TiO_2_ tube film strongly suggests that the lithium content in the film is independent of the depth of the tubes, confirming the uniform distribution of lithium in the entire Li-TiO_2_ photoelectrode. The concomitant formation of oxygen vacancies and associated Ti^3+^ ions upon the lithiation are revealed by the newly observed XPS signals of Ti 2*p* and O1*s* at the binding energies of 462.1 eV/456.6 eV (Ti 2*p*1/2 and 2*p*3/2) and 532.15 eV, respectively (Supplementary Fig. 4). This is ascribed to the reduction effect associated with the lithiation because of the lower oxidation state of Li^+^ than Ti^4+^. However, this reduction effect has a limited contribution to the PEC performance, which will be discussed in the following part.

An in-situ synchrotron X-ray diffraction (XRD) characterization of the Li-TiO_2_ electrode was used to further investigate the structure change during the electrochemical lithiation (Fig. 3d). Three main peaks (101), (004), and (200) of anatase TiO_2_ were highlighted. The (101) peak only has a slight position shift during the lithiation. An around 8 h discharging creates an additional peak at 10.1° ascribed to the new phase of the discharged product appeared^18,23,25^. At this stage, the TiO_2_ has experienced significant changes in terms of crystalline structure (weak crystallinity), phase (multiphase), and electronic status (deep reduction) due to the deep discharging process. However, at an early electrochemical stage of <6 h, the peaks assigned to anatase TiO_2_ can be clearly indexed. Meanwhile, both the (004) and (200) peaks have experienced the opposite shifts towards large and small directions, respectively. Based on Scherrer equation and peak shifts observed in Fig. 3d, the TiO_2_ lattice distances have different change extents during the discharging. The *d*_101_ keeps almost constant, whereas *d*_004_ and *d*_200_ are reduced and enlarged, respectively. The experimental results further solidify the theoretical predications of the dramatically decreased lattice constant *c* and increased lattice constant *a* in Fig. 2. The asymmetric distortions lead to the mismatch between the positive charge and negative charge centers, resulting in the formation of the dipole moment indicated by the DFT calculation. Moreover, the decrease of the intensity of all the three peaks during the lithiation (Fig. 3d) suggests the weakened crystallinity. Especially, (004) and (200) peaks almost disappear after the discharging of 10 h below the voltage platform as a result of serious lattice distortions to maintain the original TiO_2_ crystal structure with the insertion of an excess of Li ions. Moreover, the presence of lattice distortion not only leads to the formation of dipole moment but also creates additional lattice strain due to the lattice mismatch from the ideal TiO_2_ matrix. The strain can be evaluated from the XRD peak broadening according to the Williamson–Hall relationship^32,33^. The data in Supplementary Fig. 5, Supplementary Note 1, and relevant analysis suggests that the lithiation doubles the strain, further confirming the effective creation of lattice distortion in Li-TiO_2_ (Supplementary Table 2).

The obvious structure change of Li-TiO_2_ referring to TiO_2_ is characterized by high-resolution transmission electron microscopy (HR-TEM; Fig. 3e). The lattice distance (*d*) measurements via fast Fourier transfer (FFT) image (Fig. 3f and Supplementary Fig. 6) give the values of *d*_101_, *d*_200_, and *d*_004_ of samples TiO_2_ and Li-TiO_2_ in Fig. 3g (more detailed explanations in Supplementary Figs. 7 and 8). The d_101_ of TiO_2_ is similar to that of Li-TiO_2_. However, the d_004_ increases while d_200_ reduces upon the lithiation. These results are in excellent agreement with the results derived from synchrotron XRD analysis (Fig. 3d), again confirming an asymmetric change of lattices by compressing along the [004] direction and stretching along the [200] direction upon. All results presented above demonstrate that the lithiation of TiO_2_ during discharge causes the remarkable lattice distortion, which is also in consistent with the simulation results.

**Fig. 4:**
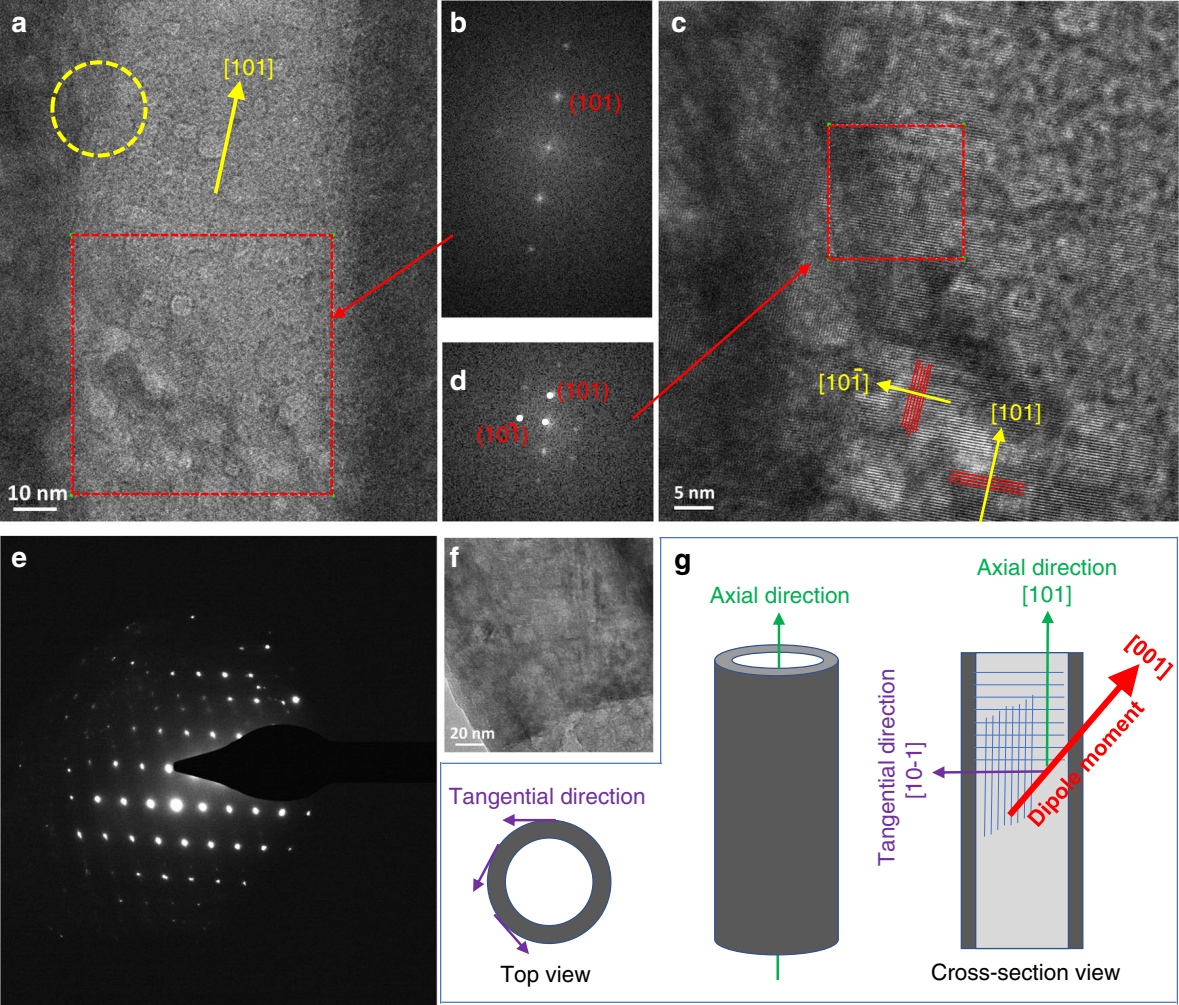
Crystallographic orientation and atomic structure of the TiO2 tube. **a** A typical TEM image of a single tube in the Li-TiO_2_ sample, prepared by cutting the tube film along the axial direction by focused ion beam (FIB, see Supplementary Fig. 9) and (**b**) FFT images of the **a** indicated with red dash square. **c** An enlarged image of the zone in **a** marked with a yellow dash circle. **d** The FFT images of the **c** indicated with red dash square. They assisted to index the lattice fringes and crystallographic orientation of the tube. **e** Selected area electron diffraction patterns recorded from a single TiO_2_ tube and (**f**) showing the corresponding part of the tube for recording the patterns. **g** Schematic of the axial and tangential directions of the tubes. The direction of dipole moment, axial crystallographic orientation, and tangential direction are illustrated in the cross-section view.

The determination of crystallographic orientation and atomic structure of TiO_2_ tubes investigated by TEM is vital to examine the dipole moment alignment. Figure 4a–d indicate that the tube has an axial crystallographic direction of [101] and tangential direction of [10–1]. Moreover, the selected area electron diffraction (SAED) patterns in Fig. 4e–f and Supplementary Fig. 9 further confirm the single crystalline-like nature of the tubes. A serial of HR-TEM images along one individual tube and the corresponded FFT and the SAED patterns (Supplementary Fig. 9 and Fig. 4e–f) confirm that the tube consists of single crystal-like texture without obvious grain boundary within a large range of sample domain of over 300 nm. The single crystal-like feature ensures the similar distortion of the TiO_6_ unit cells, resulting in well-aligned dipole along the [001] direction of the TiO_2_ crystals. As this [001] directed dipole has a significant partial vector projected to the axial direction (i.e., [101]) of the tube as illustrated in Fig. 4g, it can lead to an accumulated dipole moment along the tube axis. This inevitably causes a high system energy, which partially explains the high formation energy of the distorted TiO_2_ system predicted by the DFT.

**Fig. 5:**
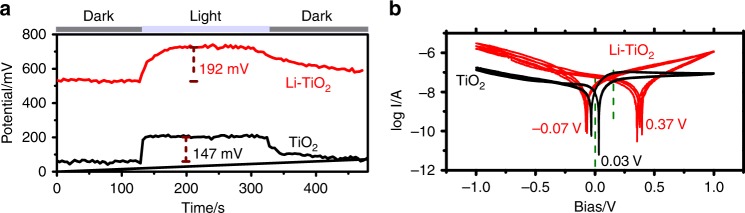
Changes of charge separation and transfer for the TiO2 electrode upon lithiation. **a** Surface potential of TiO_2_ and Li-TiO_2_ in dark and under light illumination (380 nm) measured by KPFM. **b** Electronic conductivity measured by Cycle voltammetry (CV) scanning at the rate of 50 mV s^−^^1^ for three circles. The log *I*–*V* curves instead of *I*–*V* curves are plotted for TiO_2_ (black) and Li-TiO_2_ (red). Green dotted lines represent the symmetric *V*_i_ of TiO_2_ (left) and Li-TiO_2_ (right), respectively.

The influence of the dipole moment induced by lattice distortion on the CST of the semiconductor film was firstly investigated by light-irradiated KPFM as shown in Fig. 5a. Surface potentials of the sample were recorded in dark and under light illumination, and light-induced surface potential change is known as surface photovoltage (SPV). The results of surface potential measured give two distinctive features in the samples of TiO_2_ and Li-TiO_2_: (i) Li-TiO_2_ shows much higher surface potential in dark than TiO_2_; (ii) Li-TiO_2_ also has higher SPV signal under 380 nm light illumination. The surface potential reflects the surface work function of sample, which is directly associated with the surface band bending and/or dipole. Upon lithiation, the surface potential drastically increases from 60 mV to 530 mV, indicating a remarkable decrease of surface work function by about 470 mV. The positive SPV of Li-TiO_2_ was significantly larger than that of TiO_2_ (192 vs. 147 mV). As the magnitude of SPV is intrinsically determined by the amount of separated charges in the photoelectrode^34,35^, the larger SPV suggests the accumulation of more photogenerated holes on Li-TiO_2_ surface under light illumination. These results verify that the dipole induced by the lithiation plays a critical role in promoting charge separation. Benefiting from the dipoles derived electric field in the bulk, the photo-induced electron-hole pairs can be efficiently separated, resulting in enhanced SPV. Considering the nature of lithiation induced dipoles, a higher SPV might be generated with the stronger dipole moment caused by inserting a higher amount of Li in TiO_2_ with the retained crystal structure. Meanwhile, the suppression of the charge recombination process by the dipole is indicated by a much slower recovery time of the surface potential of Li-TiO_2_ than TiO_2_ after turning off the light irradiation (Fig. 5a) as reported^36^.

The presence of a bulk electric field also facilitates the charge transfer in the materials. By assembling the electrode into a device (Supplementary Fig. 10 and Supplementary Note 2), the electronic conductivity properties of the tube films were studied. The result of the much higher current (*I*) of Li-TiO_2_ than TiO_2_ as a function of the scan bias (*V*) in Fig. 5b, Supplementary Fig. 10, and Supplementary Note 2 suggests that the charge transfer in Li-TiO_2_ film has a much lower resistance than that in TiO_2_ film. Note that, there exist two spikes in the curves of *V*-log *I* corresponding to the bias intersection (*V*_*i*_) at the *I* = 0 A points (Supplementary Fig. 10e, f). TiO_2_-based device fabricated is similar to a plane capacitor with TiO_2_ as the dielectric, which leads to a symmetric *V*_i_ (±0.3 V) centered at 0 V. For Li-TiO_2_, the center of *V*_i_ (−0.07 V, 0.37 V) is at 0.15 V. This potential shift from 0 to 0.15 V is ascribed to the built-up of electric field in the bulk of TiO_2_ film after inducing dipole by Li-ion insertion. (Detailed discussion in Supplementary Information). This positive potential suggests that the direction of electric field points to the surface from the substrate, which is definitely beneficial for the separation and transfer of photogenerated electrons and holes. (Detailed discussion in Supplementary Information). The average electric field is evaluated to be 2.1 × 10^2^ V cm^−1^ according to the measured voltage of 0.15 V and film thickness of 7 μm. Thus, there exists not only the conventional built-in electric field of ~10^4^ V cm^−1^ at surface charge layer^35^ but also an additional bulky electric field of 2.1 × 10^2^ V cm^−1^ across the film of Li-TiO_2_ tube arrays. In strong contrast, as the photoelectrodes do, pristine tube array film of TiO_2_ gives negligible electric field. The new built-in electric field is important in suppressing the charge recombination to drive the CST in the bulk Li-TiO_2_ film for enhanced PEC process^34,35^.

### Enhanced PEC performance of the lithiated TiO_2_

**Fig. 6:**
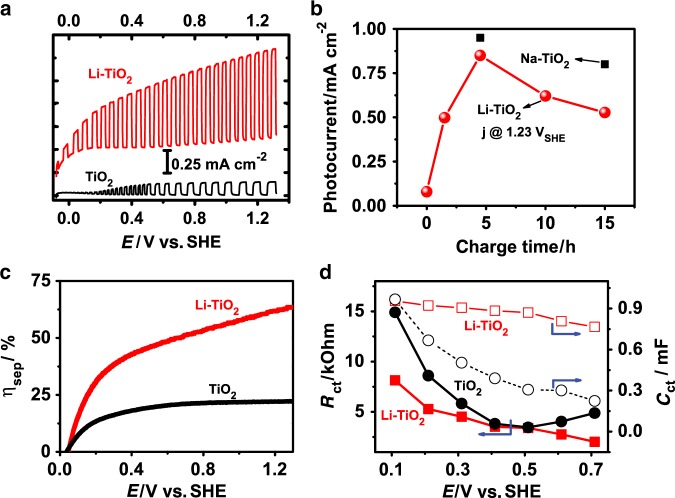
The electrochemical properties of the photoanodes. **a** The photocurrent density of (black) TiO_2_ and (red) Li-TiO_2_ under chopped light. **b** The photocurrent of TiO_2_ photoelectrode as a function of the duration of Li-ion or Na-ion insertion process. **c** Comparison of the charge separation efficiency of TiO_2_ and Li-TiO_2_ photoanodes estimated in electrolyte of 0.5 M Na_2_SO_4_ + 0.5 M H_2_O_2_ solution. **d** Charge transfer resistance (filled dot) and capability (empty dot) of TiO_2_ (black square) and Li-TiO_2_ (red circle) photoanodes.

To verify the lattice distortion effect on the PEC water splitting, the photocurrent densities of the samples were measured as a function of the bias applied. The photocurrent density of TiO_2_ photoelectrode is drastically improved from 0.14 to 1.05 mA cm^−2^ at 1.23 V vs. SHE upon Li insertion, corresponding to a 750% improvement (Fig. 6a). The incident photon-to-current efficiency (IPCE; Supplementary Fig. 11) as a function of wavelength of light irradiation consistently reveals the much higher efficiency of Li-TiO_2_ than TiO_2_ photoelectrode. The onset potential, another important parameter, has around 100 mV negative shift, which means a reduced external bias input to be provided to drive water splitting. The dependence of the photocurrent of Li-TiO_2_ photoelectrode as a function of the lithiation duration in Fig. 6b shows that all durations can apparently enhance photocurrent and the optimal duration of 5 h leads to a largest enhancement. The continuous enhancements with the duration increase before the optimal lithiation are ascribed to the increased dipole moment at the higher lithium in TiO_2_. The photocurrent decrease after the optimal duration is ascribed to the damage of the crystallinity of Li-TiO_2_ as demonstrated by in-situ XRD measurements (Fig. 3h). The charge separation efficiency (*η*_sep_) can be evaluated in the presence of hole scavenger (Fig. 6c)^4,37^. The pristine TiO_2_ electrode only has a *η*_sep_ of lower than 25 %, which is less than half of the *η*_sep_ of Li-TiO_2_. The charge transfer resistance (*R*_ct_) in the PEC process measured by electrochemical impedance spectroscopy (EIS; Supplementary Fig. 12)^38^ is depicted in Fig. 6d. The Li-TiO_2_ gives a lower *R*_ct_ than TiO_2_ in the overall potential range investigated, indicating that Li-ion insertion does promote the charge transfer process. As a consequence of the higher charge separation efficiency and lower charge transfer resistance, more charges survived take part in the surface reaction for Li-TiO_2_ and thus lead to improved capacitance (*C*_ct_). The stability test of the Li-TiO_2_ electrode (Supplementary Fig. 13) suggests a high stability of PEC water splitting in alkaline solution (NaOH pH 13.6). It is noteworthy that the Li-ion has a risk of being etched out from the structure under low pH solution (e.g., Na_2_SO_4_ pH 6.5), leading to a considerable decay of the photo-response.

Considering the fact that lithiation changes both the structure (lattice distortion) but also the composition of TiO_2_ (reduced oxidation state of Ti and inserted Li), it is necessary to identify their individual contributions to PEC performance, to get how much the lattice distortion-associated bulk electric field improves the PEC activity. Injection of lithium insertion-accompanied electrons into TiO_2_ leads to a prominent reductive effect as evident by the formed oxygen vacancies and related Ti^3+^ (Supplementary Fig. 4). The reduced Ti species might have the functions of extending the light harvesting of TiO_2_ and improving the carrier concentration. By comparing the IPCE curves (Supplementary Fig. 11), it is clear that TiO_2_ and Li-TiO_2_ photoelectrodes have a similar light response range with an IPCE threshold of around 420 nm; thus, the influence of extended light harvesting on the PEC performance can be safely ruled out as reported^39^. To further investigate the influence of the carrier concentration change on the photocurrent enhancement, the TiO_2_ electrode is reduced (r-TiO_2_) electrochemically by passing the same amount of charges as the discharging process (Supplementary Fig. 3)^40^. The resultant r-TiO_2_ electrode with a similar free carrier concentration to that of the Li-TiO_2_ electrode, determined by Mott–Schottky (M-S) curves (Supplementary Fig. 14 and Supplementary Note 3) also gives an enhanced photocurrent, which is, however, only half of that of Li-TiO_2_ photoanode (Supplementary Fig. 15). In addition, both the r-TiO_2_ and Li-TiO_2_ photoanodes show obvious dark current that is not observed in pristine TiO_2_ photoanode, suggesting that the dark current might be due to the greatly improved electronic conductivity of the TiO_2_ films with a high concentration of charge carriers. These results suggest that the increased carrier concentration does make a contribution to the photocurrent enhancement, to some extent, but cannot totally account for the drastic photocurrent increase of Li-TiO_2_ photoelectrode. The structure change should make a substantial contribution to the photocurrent.

The influence of carrier concentration change on the photocurrent of Li-TiO_2_ was further evaluated by comparing the PEC performance of lithiated TiO_2_ photoelectrodes with high and low concentrations of charge carriers. The latter electrode was obtained by heating the former one in air. To avoid the possible influence of Ti substrate oxidation during heating process on the PEC performance, the films of rutile TiO_2_ nanorod arrays on fluorine doped tin oxide (FTO) glass substrates (TiO_2_ | FTO) were prepared for this purpose. Lithiation of the TiO_2_ | FTO photoelectrode (see Supplementary Experiments for details) also significantly improves its photocurrent (Supplementary Fig. 16a), demonstrating the generic feature of the lithiation strategy in improving the PEC performance of TiO_2_. Annealing the lithiated TiO_2_ | FTO (Li-TiO_2_ | FTO) photoelectrode in air led to the color change of the film of Li-TiO_2_ | FTO photoelectrode from light blue to white (a-Li-TiO_2_ | FTO; Supplementary Fig. 16a, b). The carrier concentration of a-Li-TiO_2_ | FTO decreases to the similar level to pristine TiO_2_ | FTO due to the elimination of oxygen vacancies and related defects as shown in Supplementary Fig. 16c. The PEC performance of a-Li-TiO_2_ | FTO is still much better than that of the pristine TiO_2_ | FTO (Supplementary Fig. 16d), further confirming the significant role of lattice distortion induced by Li-ion insertion. The possible contribution from the Li substitution of Ti in TiO_2_ (i.e., Li doping effect) was excluded using the Li-doped TiO_2_ | FTO photoelectrodes, which exhibited lower photocurrent to that of pristine TiO_2_ (Supplementary Fig. 17).

The positive effect of lithiation treatment in different-phase (anatase, rutile) TiO_2_ photoelectrodes suggests that the lithiation is a generic strategy in tuning the physiochemical property of TiO_2_ regardless of its phase or morphology. Furthermore, our preliminary investigation also reveals that the inserted ion could be extended to other metal ions using a similar strategy. Sodium-ion (Na^+^), which could be inserted into the TiO_2_ lattices in the sodium-ion battery (SIB), is used to modify TiO_2_ using the same method. The Na-TiO_2_ photoelectrode obtained also shows a remarkable improvement of the photocurrent (Fig. 6b), clearly indicating the generic feature of the controlled ion-insertion-induced driving force in promoting the CST in the bulk photoelectrodes.

## Discussion

A concept of creating electric field in the bulk of TiO_2_ photoelectrode by intentionally inducing lattice distortion via controllable lithiation process is proposed and validated. The bulk electric field with an average strength of 2.1 × 10^2^ V m^−1^ throughout the TiO_2_ film was built up by the aligned dipole moment produced in the distorted TiO_6_ unit cells. Benefitting from this strong built-in electric field, the photovoltage and CST efficiency of TiO_2_ photoelectrode were drastically enhanced, resulting in an over 750% improvement of photocurrent density and over 100 mV negative shift of the onset potential in Li-TiO_2_ compared with that of the pristine photoelectrode. This work highlights the significance of bulk electric field in promoting PEC process. The idea inspired from the discharge/charge processed in rechargeable ion battery might be a very promising approach to precisely tune the microstructure of the semiconductor photoelectrodes for achieving high solar conversion efficiency.

## Methods

### TiO_2_ electrode fabrication

Ti foil supported TiO_2_ tubes (TiO_2_ | Ti) are fabricated by an anodization method. Typically, a piece of Ti foil was anodized at 60 V (D.C.) for 3 h in 0.25 wt % NH_4_F solution of the mixed solvent of ethylene glycol and water (5% in volume). Then the electrode was annealed at 450 °C (the ramping rate of 5 °C min^−1^) for 3 h in a muffle oven to achieve pristine TiO_2_ electrode.

### Lithiation of the TiO_2_ photoelectrode

The prepared TiO_2_ | Ti and TiO_2_ | FTO photoelectrodes were directly assembled as the cathodes in the traditional LIBs or SIBs. Typically, the anode is the lithium or sodium. The electrolyte is 1 M LiPF_6_ dissolved in propylene carbonate (PC) or 1 M NaClO_4_ in PC. The glass fiber (What-man) is used as the separator.

### DFT calculations

The lattice parameters and the atomic structures of anatase TiO_2_ and Li_0.5_TiO_2_ were fully relaxed by using DFT calculations, which were implemented in the Vienna ab initio simulation package with the projector augmented wave pseudopotential^41–43^. Plane-wave basis set was used with a cutoff energy of 500 eV. The convergence threshold for the energy and residual force are 10^−4^ eV and 0.01 eV/Å, respectively. The Monkhorst-pack *k*-point is 5 × 5 × 2 in the Brilloin zone during the relaxation^44^. Exchange correlation functional was modeled by using generalized gradient approximation with the form of the Perdew–Burke–Ernzerhof^45^. The Hubbard U was adopted to the Ti atoms with the value of 4.2 eV.

### Kelvin probe force microscopy measurements

KPFM based on non-contact atomic force microscopy was employed for surface potential imaging, which measures the contact potential difference between the probe and sample. We use an amplitude-modulated mode of KPFM in lift mode. For each scan, a scanning capacitance microscopy-platinum/iridium tip Pt/Ir-coated conductive probe passes over the surface twice. On the first pass, a conductive probe is close to the sample surface, surface morphology and phase images can be obtained. On the second pass, the cantilever is held at 50 nm of tip-sample distance and the tip scans the same trajectory of the first scan to record surface potential. The scan rate is 0.5 Hz.

For KPFM measurements under illumination, the sample was illuminated with home-built Xenon lamp focusing system. The SRSPS setup was equipped with a KPFM unit. The monochromatic light split from a 500 W Xenon lamp by grating (Thorlabs) was used to excite sample. Surface potential signals were logged in lock-in amplifies to get SPV. Details on the experimental setup and descriptions of the experimental condition were given in recent work^46^. All measurements were tested at ambient conditions.

### Structure characterization

The morphology and crystal lattice of electrodes was imaged with field-emission SEM (JEOL JSM 7001 F) at 20 kV, HR-TEM and SAED was conducted at 200 kV (FEI, Technai 20 F), respectively. The cross-sections of the TEM samples were prepared by focused ion beam thinning (FEI SCIOS). Raman spectrum was acquired on Renishaw Micro-Raman Spectroscopy System at the wavelength of 514 nm. The XPS (Kratos Ultra) was measured for chemical analysis using a mono Al Kα X-ray source. XPS measurement was tested by Kratos Ultra. In-situ synchrotron XRD patterns (high resolution) were collected in transmission mode on powder diffraction beamline of Australian Synchrotron via the MY THEN microstrip detector and Si (111) monochromator (at the wavelength of 0.8265 Å). The 2*θ* zero-error were determined via a standard 0.3 mm capillary (a LaB 6/Si mixture using transmission geometry). Corresponding battery test was conducted on a Neware electrochemical tester.

### (Photo)electrochemical characterization

All the (photo)electrochemical characterization was carried out in an electrolyte of 0.5 mol L^−1^ Na_2_SO_4_ (adjust to pH 7) in a three-electrode system with Pt as the counter-electrode and Ag/AgCl electrode (with saturated KCl solution) as the reference electrode. The light power is adjusted to 100 mW cm^−2^ via control the power of a Xenon lamp (Newport) equipped with AM 1.5 G filter. The current was recorded by linear sweep voltammetry in an electrochemical work station (CHI 660D) within the range of −0.7 to 0.7 V vs. Ag/AgCl and the photoelectrodes were illuminated from the semiconductor/electrolyte side. The EIS and M-S curves were measured by Ivium potentiostat. For EIS, it was recorded under a serial of applied bias in a frequency range of 10^−1^–10^5^ Hz with a potential amplitude of 10 mV. In addition, the M-S curves were measured at the frequency of 10^3^ Hz within a potential range of −0.8 to 0 V vs. Ag/AgCl.

The charge separation efficiency was evaluated with the presence of H_2_O_2_ as scavenger. Typically, the photocurrent was measured with (*j*_H2O2_) the presence of H_2_O_2_ scavenger.

The potential (*E*_Ag/AgCl_) referred to Ag/AgCl can be converted into the standard hydrogen electrode (*E*_SHE_) scale with the Nernst Eq. (1):

1 $$E_{SHE} = E_{Ag/AgCl} + 0.059 \times pH + 0.197$$

## Supplementary information

Supplementary Information

## Data Availability

The authors declare that all the relevant data within the paper and its Supplementary Information file or from the corresponding author upon reasonable request. The source data of Figs. 2g, 3c, 3d, 3g, 5, and 6, and Supplementary Figs. 1c, d, 3, 4, 5, 10a, b, 11, 12, 13, 14, 15, 16c, d, and 17 are provided as Source Data file.

## Source data

Source Data
