# Supplementary material for: Lattice distortion induced internal electric field in TiO2 photoelectrode for efficient charge separation and transfer
Source: Nat Commun. 2020 May 1;11:2129. doi: 10.1038/s41467-020-15993-4 (PMC7195485; doi:10.1038/s41467-020-15993-4)
Supplement: Supplementary file 1 — Supplementary Information [file 41467_2020_15993_MOESM1_ESM.pdf]

Supplementary Information

Lattice distortion induced internal electric field in TiO_2 photoelectrode for efficient charge separation and transfer

Hu, *et al.*

Supplementary Figures

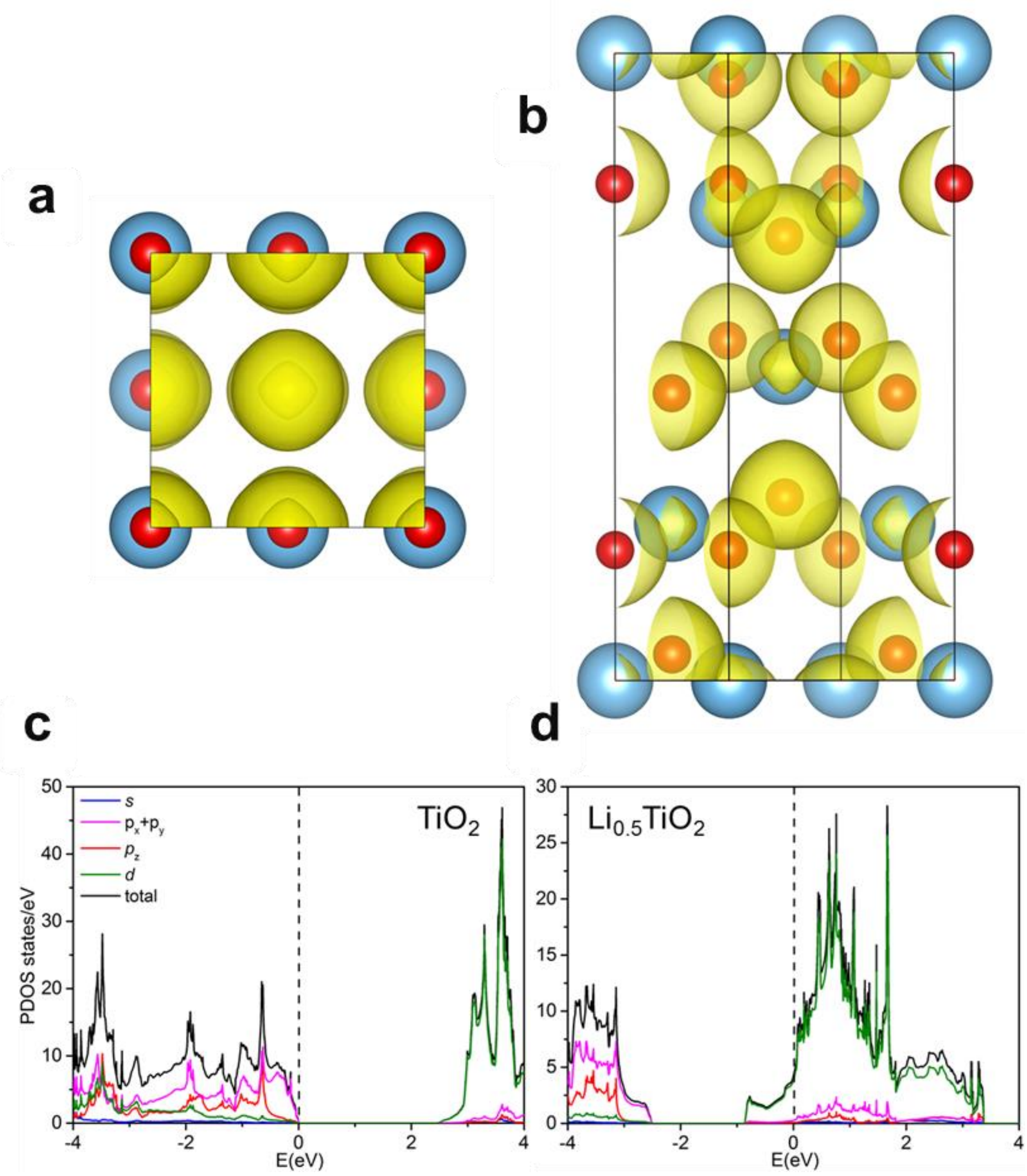

Supplementary Figure 1 | Electron distribution and density of states. (a, b) top view and side view of the electron density distribution of the pristine TiO_2 system. The blue and red balls represent Ti and O atoms, respectively. Yellow surface represents the electron distribution. (c, d) Density of states (DOS) (DOS on specified orbitals) of TiO_2 and $Li_{0.5}TiO_2$ at the DFT level. The Fermi level is at zero energy and represented by dashed lines.

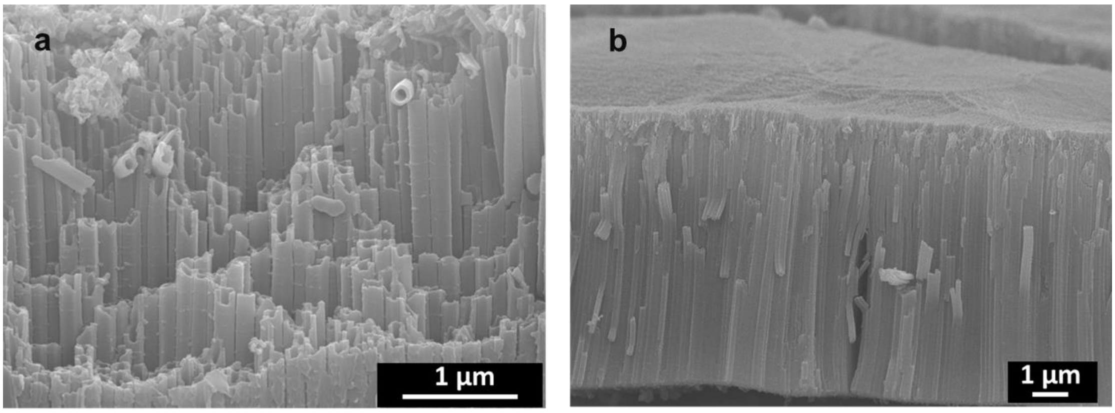

Supplementary Figure 2 | SEM images of pure TiO_2. (a) Top-view and (b) cross section-view SEM images of pure TiO_2 tubes supported on Ti foil.

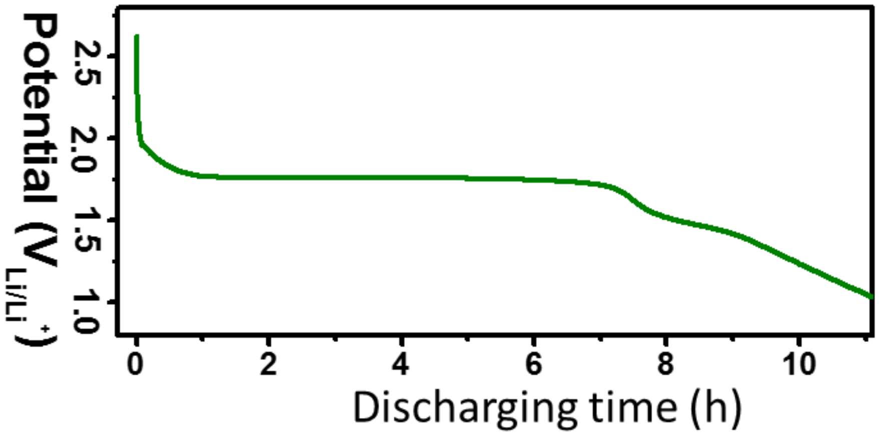

Supplementary Figure 3 | Discharge curve of Li-TiO_2 nanotube array film based battery under the current of 0.03 mA (around 0.08 C).

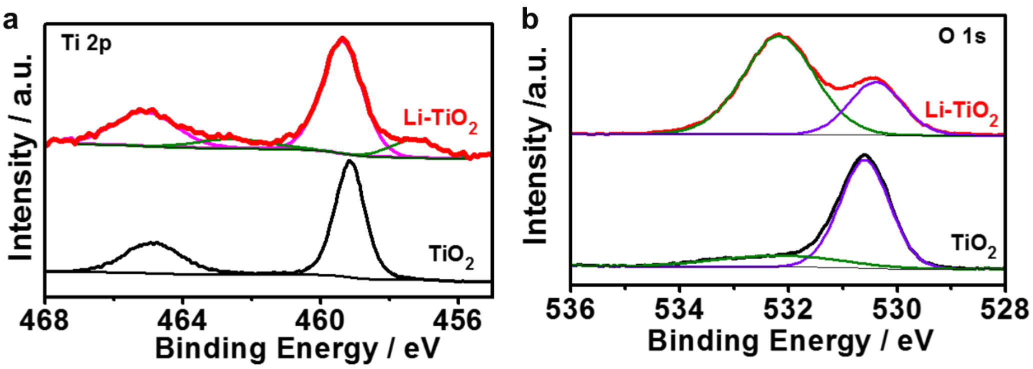

Supplementary Figure 4 | X-ray photoelectron spectroscopy spectra of samples. (a) Ti 2p and (b) O 1s for the pristine TiO_2 (black line) and Li-TiO_2 (red line). In addition to two dominant peaks at 465.1 eV (Ti 2p1/2) and 459.3 eV (Ti 2p3/2) associated with Ti^{4+} in Li-TiO_2, two additional peaks at 462.1 eV (Ti 2p1/2 level) and 456.6 eV (Ti 2p3/2 level) can be assigned to Ti^{3+}. In O 1s spectrum of Li-TiO_2, the strong peak at 532.4 eV can be assigned to oxygen vacancies, which is typically observed for reduced TiO_2.[1]

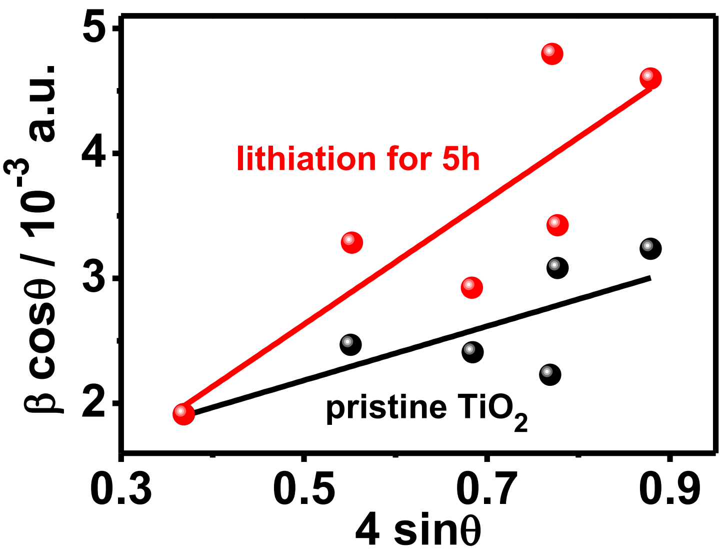

Supplementary Figure 5 | The Williamson-Hall (W-H) relationship for the pristine and 5 h-lithiation treated TiO_2 samples. It is derived from the full-width Half Maximum (FWHM, β) and the diffraction peaks (2θ) of TiO_2. A least squares (R^2) method is used to fitting the original data (dots) with a linear relationship (solid line)

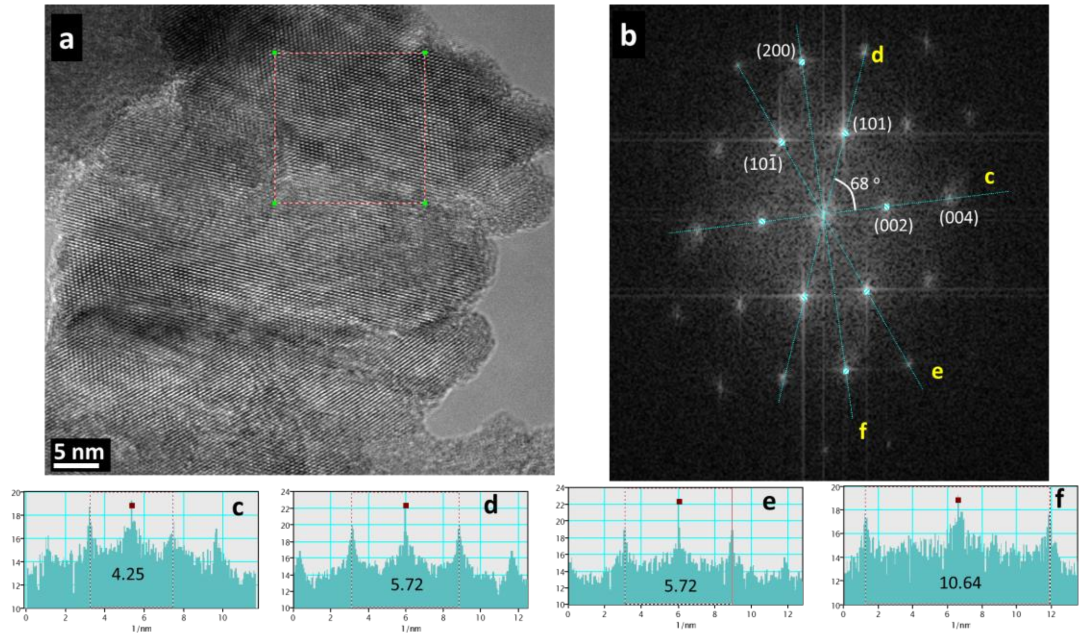

Supplementary Figure 6 | HR-TEM image and FFT analysis of TiO_2 nanotubes. The selected area with accurate focus in (a) is processed with fast Fourier transfer (FFT) as indicated in (b). According to the angle and distance between the diffraction points, the facets in the FFT image can be indexed. The lattice distance is calculated based on the distance in FFT images (c~f), where d1, d2, d3, and d4 equal to 0.471 nm (2/4.25), 0.350 nm (2/5.72), 0.350 nm (2/5.72), and 0.188 nm (2/10.64). In the meanwhile, d_{004}, d_{101}, and d_{200} was 0.238 nm, 0.352 nm, and 0.189 nm in anatase TiO_2 (PDF#21-1272). Thus, it is concluded that the facets in (1), (2) and (4) belong to {002}, {101} and {200} as shown in (b).

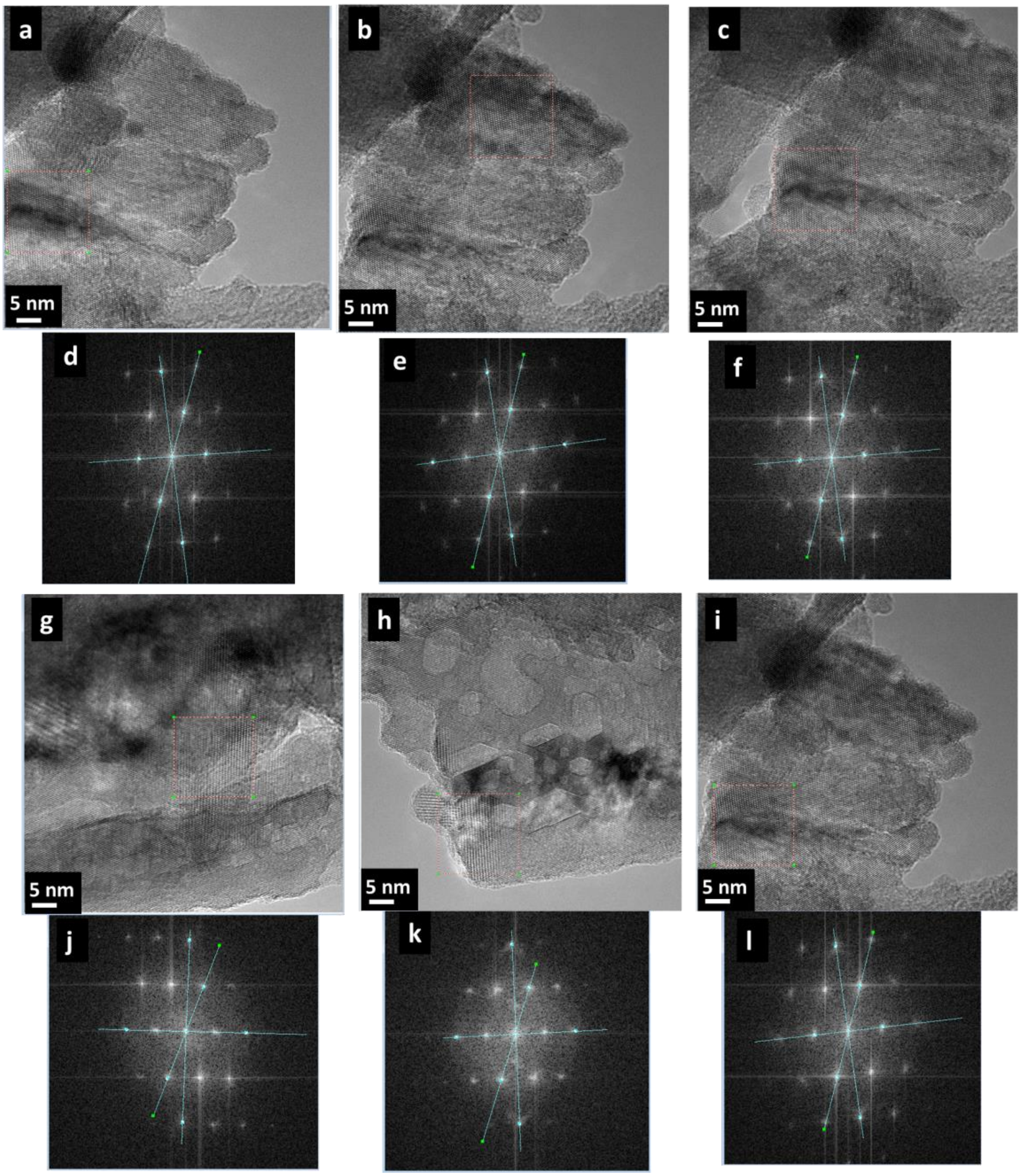

Supplementary Figure 7 | HR-TEM image and FFT of TiO_2. According to these TEM images (a, b, c, g, h, i) and the corresponded FFT images (d, e, f, j, k, l), the lattice distance of d_{101}, d_{200}, and d_{004} is 0.348±0.0007 nm, 0.187±0.0006 nm, and 0.249±0.0199 nm, respectively.

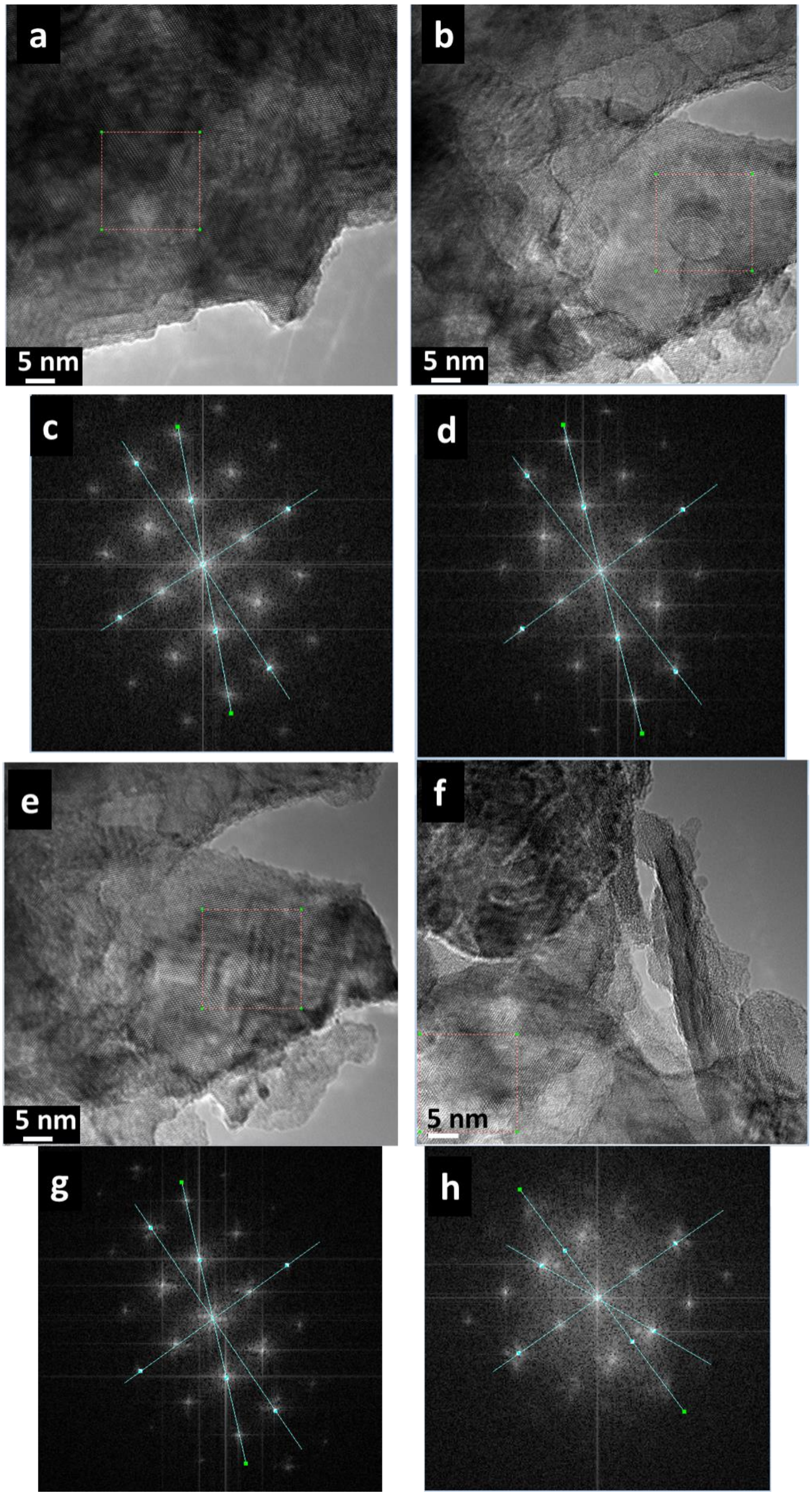

Supplementary Figure 8 | HR-TEM image and FFT analysis of Li-TiO_2. According to these TEM images (a, b, e, f) and the corresponded FFT images (c, d, g, h),, the lattice distance of d_{101}, d_{200}, and d_{004} is 0.348±0.0003 nm, 0.189±0.0012 nm, and 0.231±0.0016 nm, respectively.

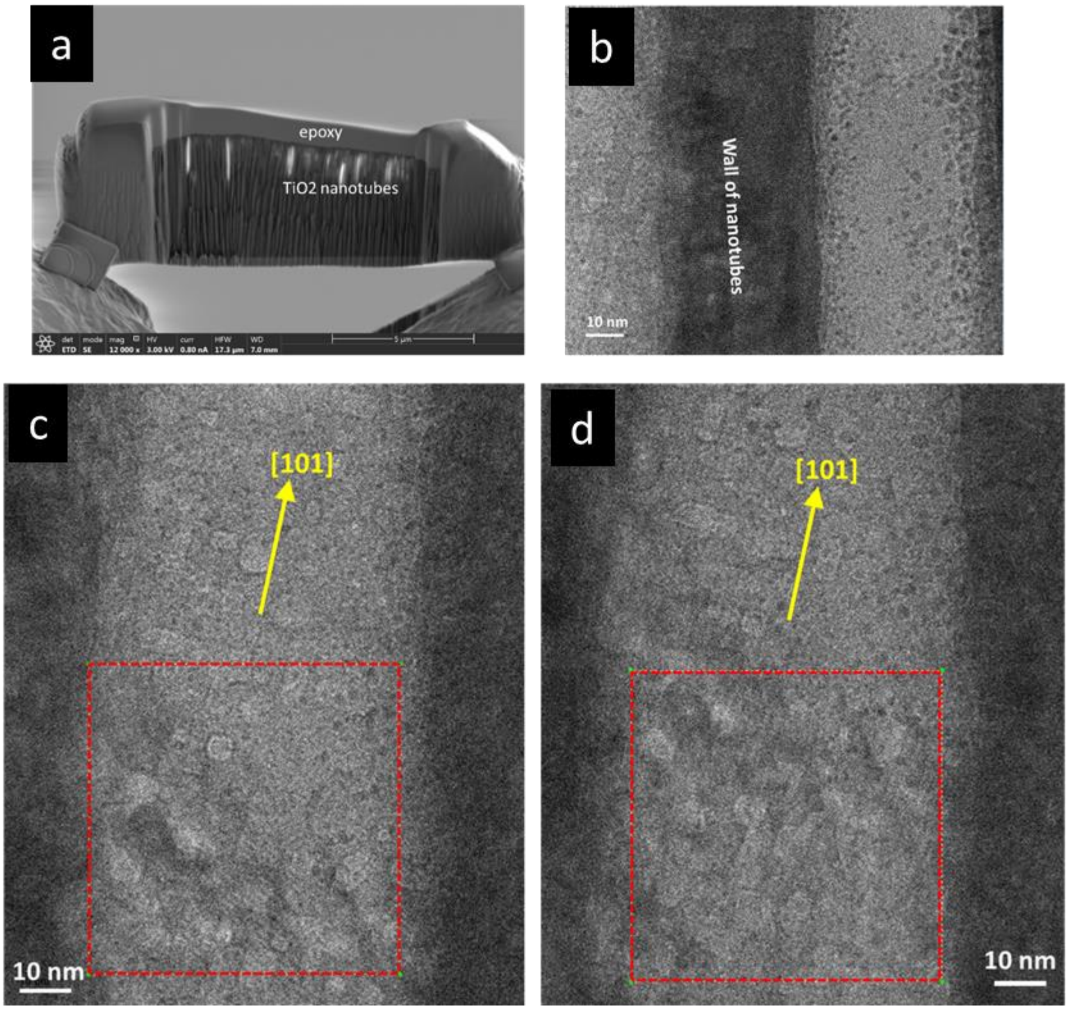

Supplementary Figure 9 | TEM images of Li-TiO_2 tubes. (a) TEM image of TiO_2 tube photoelectrode cut by focused ion beam (FIB). (b) HR-TEM image of the wall of two adjacent tubes. (c) and (d) The HRTEM images of a single TiO_2 tube at two adjacent zone with a total length of *ca*. 300 nm.

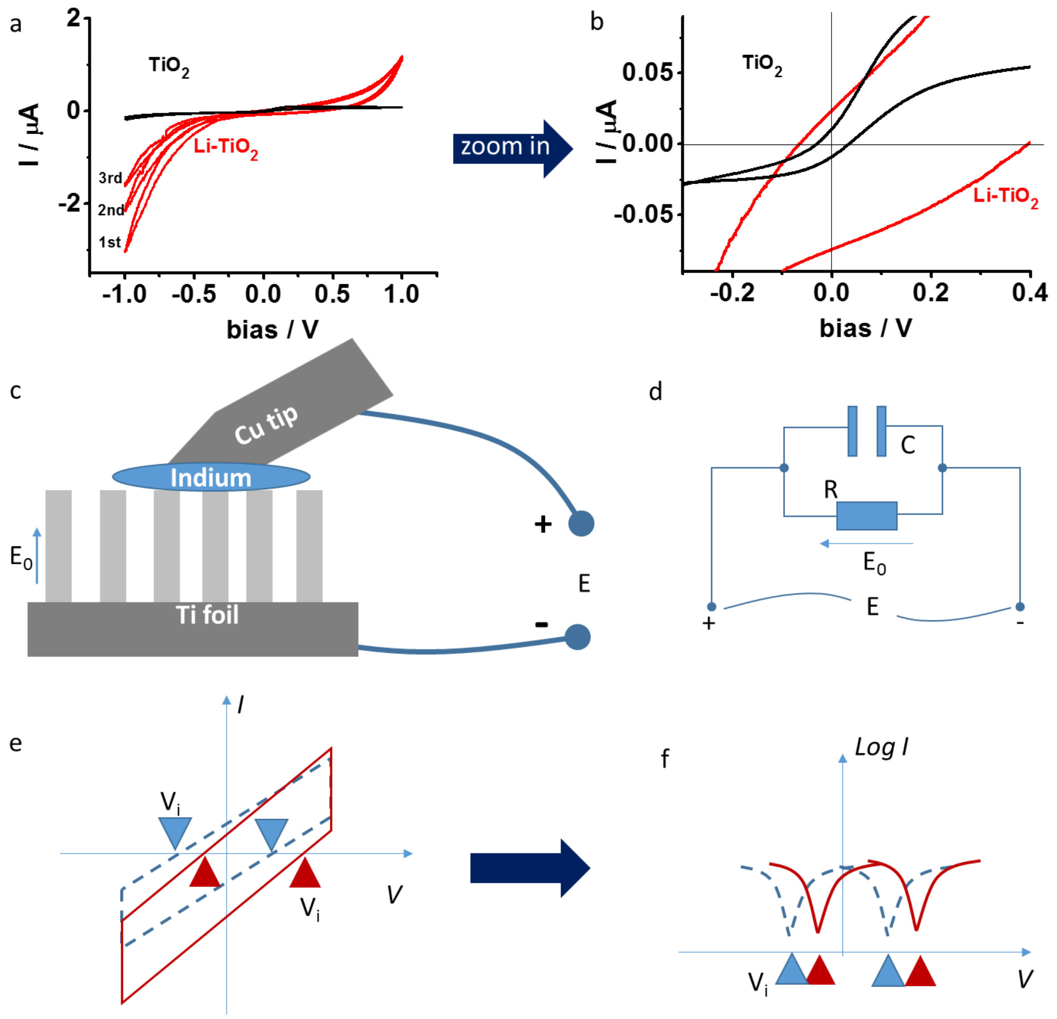

Supplementary Figure 10 | *I-V* characterization of the electrode. (a) cyclic voltammetry (CV) scan of the devices fabricated from (black line) TiO_2 and (red line) Li-TiO_2. (b) zoom-in image of (a) to indicate the intersection voltage (V_i) at I = 0. (c) schematic of the device used for CV measurement. (d) theoretical equivalent circuit of (c). (e) ideal *I-V* response during CV scan with (red line) and without (blue line) a constant bias (E_0) on the resistor (R) according to (d). (f) The plots of log *I* as a function of V. The intersection voltage (V_i) in (e) is highlighted as sharp spikes in (f).

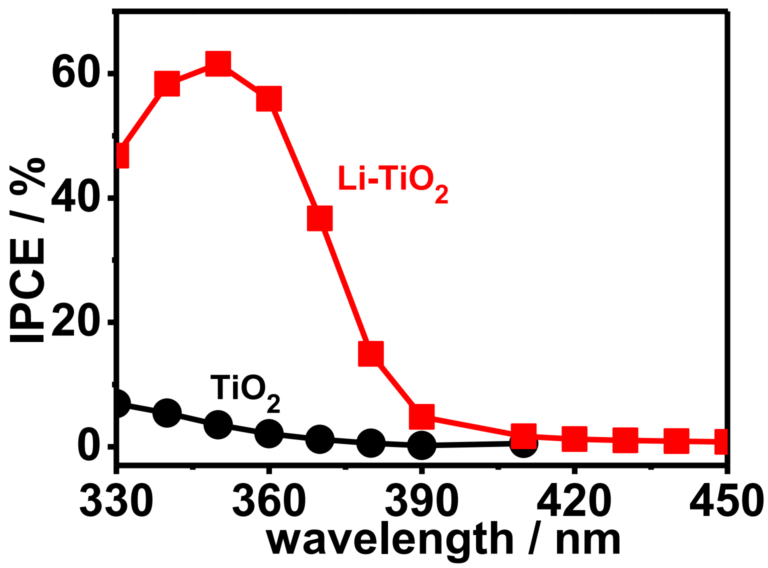

Supplementary Figure 11 | The incident photon-to-current efficiency (IPCE) of TiO_2 and Li-TiO_2 photoelectrodes. The Li-TiO_2 photoanode shows a threshold at the wavelength of around 420 nm, with is in consistent with the bandgap of TiO_2. The increased light absorption beyond 420 nm caused by the lithiation-induced reduction effect makes negligible contribution to the photocurrent response.

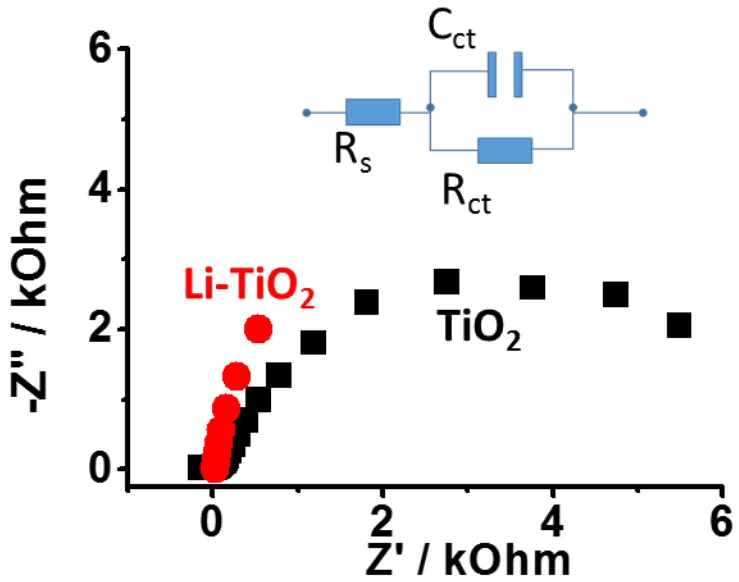

Supplementary Figure 12 | Electrochemical impedance spectroscopy (EIS) of (black line) TiO_2 and (red line) Li-TiO_2 and the equivalent circuit used for fitting.

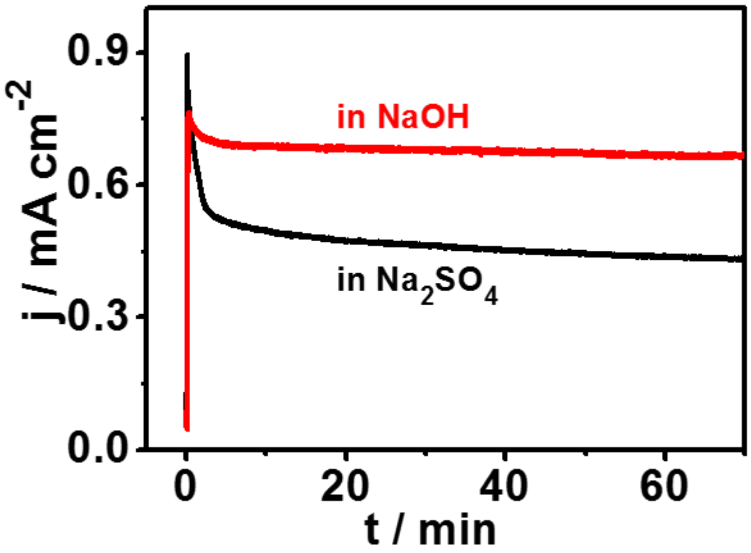

Supplementary Figure 13 | The photocurrent stability tests in 1 M NaOH solution (pH = 13.6) and 0.5 M Na_2SO_4 (pH = 7) at 1.0 V *vs* SHE. The photocurrent keeps a good stability in NaOH solution for a whole test but experiences a rapid decrease at the first 5 minutes and then gradual decrease in Na_2SO_4 solution.

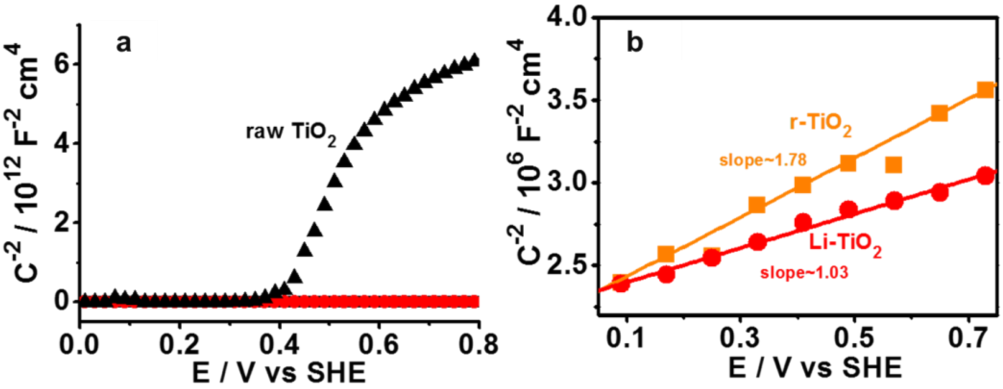

Supplementary Figure 14 | Mott-Schottky (M-S) curves of samples. (a, b) M-S curves of (black line) TiO_2, (red line) Li-TiO_2 and (orange line) reduced TiO_2 (r-TiO_2) TiO_2 nanotube photoanodes.

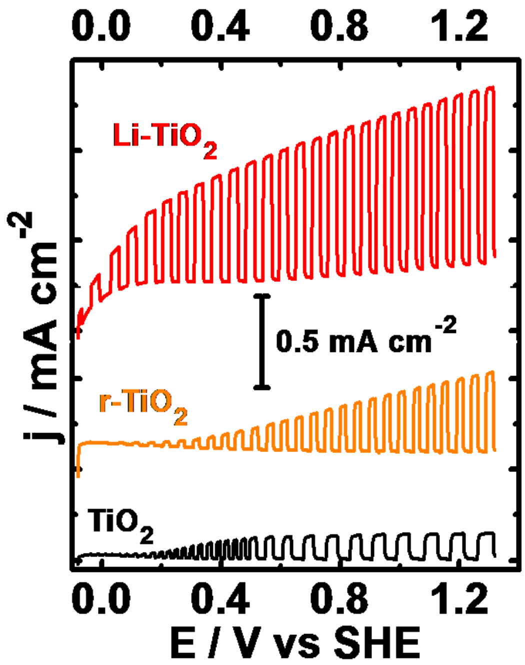

Supplementary Figure 15 | The photocurrent of (black line) pristine TiO_2, (orange line) reduced TiO_2, and (red line) Li-TiO_2. For the r-TiO_2 electrode, electrochemical reduction at -1.6 V vs Ag/AgCl was carried out deliberately. The amount of charge used to reduce TiO_2 is the same (0.08 C) as that passed through Li-TiO_2 during the discharging. In this way, the amount of reduced Ti species is anticipated to be close in r-TiO_2 and Li-TiO_2 electrodes, as indicated by the Mott-Schottky curves in Fig. S10.

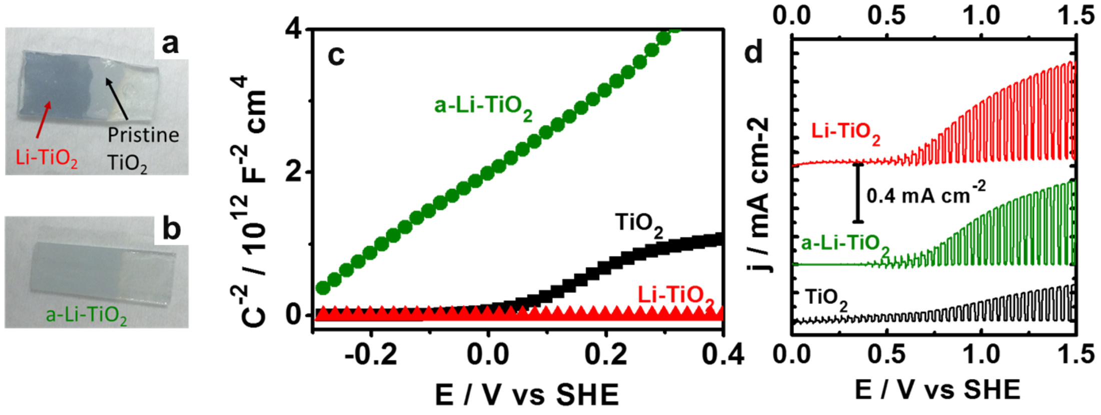

Supplementary Figure 16 | Performance based on TiO_2|FTO photoelectrodes. (a, b) Optical images of pristine TiO_2 nanorod electrode, lithiated TiO_2 nanorod photoanode (Li-TiO_2|FTO) and annealed Li-TiO_2|FTO (a-Li-TiO_2|FTO). The pristine TiO_2|FTO and a-Li-TiO_2|FTO have nearly white color, and the Li-TiO_2|FTO has blue color. (c) Mott-Schottky curves of the TiO_2|FTO, Li-TiO_2|FTO, and a-Li-TiO_2|FTO photoanodes and (d) photocurrent response curves of three photoelectrodes under 1 sun simulated light. Based on the slope of M-S curves, the charge carrier concentration of Li-TiO_2 was determined to be much higher than that of TiO_2 due to the reductive effect of lithiation. The carrier concentration of a-Li-TiO_2 is comparable to that of TiO_2. The Li-ions accommodated in the a-Li-TiO_2 can cause lattice distortion. Therefore, comparison of the photocurrent density of a-Li-TiO_2|FTO, Li-TiO_2|FTO and TiO_2|FTO in (d) clearly indicates the substantial role of lattice distortion in enhancing photocurrent.

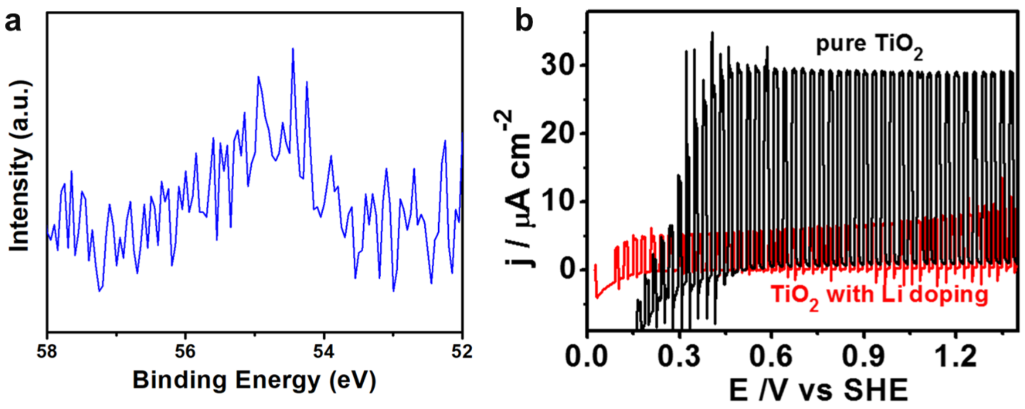

Supplementary Figure 17 | Characterization of Li doped TiO_2 reference sample. (a) XPS spectrum of Li 1s in Li doped TiO_2 reference sample and (b) photocurrent-bias curves of undoped and Li doped TiO_2 photoanodes.

Supplementary Tables

Supplementary Table 1 | The charge distribution around each atom in $Li_{0.5}TiO_2$ lattice cell.

		X	Y	Z	**CHARGE***
1	Ti	3.9829	3.9910	4.7682	2.0689
2	Ti	3.9829	1.9925	7.1951	2.1479
3	Ti	1.9421	1.9927	9.1435	2.1199
4	Ti	1.9407	3.9905	2.3530	2.0688
5	O	1.9420	3.9911	4.4369	7.2128
6	O	3.9824	3.9914	6.7435	7.0801
7	O	3.9832	1.9923	4.9819	7.2215
8	O	1.9421	1.9924	7.1291	7.1323
9	O	3.9830	1.9926	9.2097	7.1261
10	O	1.9409	1.9917	2.1392	7.2437
11	O	1.9400	3.9899	0.3776	7.0762
12	O	3.9818	3.9909	2.6846	7.2107
13	Li	3.9802	1.9914	2.0234	0.1457
14	Li	1.9427	1.9923	5.0950	0.1455

*It is the average valence electron number around each atom in the lattice cell schemed in Fig. 2b. According to the results, there are 0.145 electrons around Li atoms on average, suggesting that each Li atoms will donate 0.845 electrons.

Supplementary Table 2 | The W-H relationship for pristine TiO_2 and the Li-TiO_2

	Slope	intercept	R^2
Pristine TiO_2	0.00217	0.00110	0.667
5 h lithiated TiO_2 (Li-TiO_2)	0.00498	0.00014	0.664

Supplementary Notes

Supplementary Note 1. In Supplementary Figure 5, according to the Williamson-Hall relationship, the broadening of peaks can be expressed as Supplementary Equation 1:[2]

$$\beta \times \cos\theta = \frac{k \times \lambda}{D} + 4\varepsilon \times \sin\theta \qquad (1)$$

Where k is a constant, λ is the irradiation wavelength, D is the particle diameter and ε is the lattice strain. Accordingly, the $\beta \times \cos\theta \sim 4 \times \sin\theta$ should give a linear relationship with the slope of strain (ε). Based on the diffraction peaks of (101), (004), (200), (211), (105) and (204) from the synchrotron XRD, the W-H relationship for pristine TiO_2 and the one after 5 h lithiation can be fitted within Table S1.

Supplementary Note 2. In Supplementary Figure 10, an indium particle is placed on the top of photoelectrode to enhance the contact between the tip and semiconductor film, and to avoid the short-circuiting. Soft indium particle can easily be pasted on the electrode top so it can work as an intermediate layer to enhance the contact between the tip and nanotube film. Moreover, the solid-state nature of indium prevents its penetration through the tubes to reach the bottom Ti foil, causing short-circuit.

The device in (c) is similar to a physical plane capacitor with a metal|dielectric|metal (Ti|TiO_2|In) structure. So a parallel capacitor with a resistor in (d) is used to simulate the equivalent circuit. In this case, we can predict the *I-V* relationship at the scan rate of $\pm v$ during the CV scan as Supplementary Equation 2:

$$I=(V-E_0)/R \pm v C \qquad (2)$$

In Supplementary Equation 3, (V-E_0)/R is the current passed through the resistor, which is independent of the scan rate. The slope of *I-V* curves is determined by the resistance (R) of the film. The $\pm vC$ is the current passed through the capacitor, which is determined by the scan rate (v) and capacitance (C), but independent of the applied voltage (V). E_0 is voltage created by dipolar in the bulk of TiO_2.
According, the intersected voltage (V_i) can be expressed as:

$$V_i = E_0 \pm vC/R \qquad (3)$$

For pure TiO_2, E_0 is null, therefore, V_i should be $\pm vC/R$, which is a mirror symmetry at V=0. This result is constant with the result in (b) and Figure 5b (V_i = ±0.03 V).
For Li-TiO_2, the symmetry axis shows a positive shift (V_i = -0.07, 0.37 V), suggesting that there is a positive E_0 generated in the device. Considering the configuration of the device and measurement, it is concluded that there should be an electric field directed towards the surface from the bottom as indicated in (c).

Supplementary Note 3. In Supplementary Figure 14, according to the *M-S*

Supplementary Equations 4 and 5:[3]

$$C^{-2} = \frac{2}{q\varepsilon N_d}\left(E - E_{fb} - \frac{k_B T}{q}\right) \quad (4)$$

$$N_d = \frac{2}{q\varepsilon}\left({dC^{-2}}/{dE}\right)^{-1} \quad (5)$$

Where q is the elemental charge, ε is the dielectric constant, k_B is the Boltzman constant, E_{fb} is the flat band potential, T the temperature in kelvin, N_d is the carrier concentration.

The carrier concentration can be evaluated from the slope of M-S curves (${dC^{-2}}/{dE}$).

Upon lithiation, the N_d is significantly improved due to the reductive effect. For the electrochemically reduced r-TiO_2, the amount of charge passing the electrode is the same as that passing Li-TiO_2. Thus, r-TiO_2 and Li-TiO_2 were reduced at a similar extent and have comparable concentrations of charge carriers as indicated by the slope of M-S curves in Supplementary Figure 14 (b).

Supplementary Methods

Synthesis. Rutile TiO_2 photoelectrodes grown on FTO glass substrates are prepared by a hydrothermal method.[4] Typically, 1.0 mL of titanium butoxide (TBT, Sigma-Aldrich) was dissolved in a mixed solution containing 20 mL of hydrochloric acid (Sigma-Aldrich) and 30 mL of distilled water. A piece of clean FTO glass substrate was placed into the solution vertically in an autoclave with the Polytetrafluoroethylene (PTFE) line. The hydrothermal reaction was carried out at 180 ^oC for 2 h. After cooling down to room temperature, the TiO_2|FTO electrode was cleaned and further annealed at 450 ^oC (the ramping rate of 5 ^oC min^{-1}) for 2 h. TiO_2 in the electrode has the nanorod morphology. The lithiation of TiO_2|FTO photoelectrode was conducted following the same procedures as for the TiO_2 nanotube photoelectrode. The resultant electrode is denoted as Li-TiO_2|FTO photoelectrode. In order to quench oxygen vacancies caused by the lithiation, the Li-TiO_2|FTO photoelectrode was then annealed in air at 450 ^oC (the ramping rate of 1 ^oC min^{-1}) for another 2 h and the sample obtained is denoted as a-Li-TiO_2|FTO photoelectrode.

The Li doped TiO_2|FTO photoelectrodes were prepared by spin coating.[5-7] 0.2 M TBT ethanol solution was used as precursor and LiAc was dissolved in it to make the Li/Ti molar ratio of 0.45 (the same ratio in Ti foil supported Li-TiO_2 photoelectrode). The film was fabricated at a spin rate of 1000 rpm for 1 min with 0.1 mL of precursor solution dispersed on an FTO glass. After spin coating, the film was heated at 120 ^oC for 5 minutes on a hot plate. The procedure was repeated 10 times to increase the film thickness. In the end, the film was annealed at 450 ^oC for 3 h in a muffle oven to achieve the anatase phase.

The incident photon-to-current efficiency (IPCE) test The IPCE was obtained using an Oriel Cornerstone 260 1/4 m monochromators with a 300 W Oriel Xenon lamp as the simulated light source. The light power was calibrated with a standard Si diode. The power density at a specific wavelength was measured by a Newport 1918-c power meter. The IPCE can be calculated according to following Supplementary Equation 6.[3]

$$\text{IPCE} = \frac{j\ (\text{mA cm\^{}-2}) \times 1239.8\ (\text{V}\cdot\text{nm})}{\lambda\ (\text{nm}) \times I\ (\text{mW cm\^{}-2})} \times 100\% \qquad (6)$$

j is the photocurrent density (mA cm^{-2}) measured from the electrochemical workstation, λ refers to the incident light wavelength (nm), and I is the light density measured at a specific wavelength (mW cm^{-2}).

DFT calculation of the formation energy The formation energies of Li_nTiO_2 were defined as Supplementary Equation 7:

$$\Delta E = (E_{Li_nTiO_2} - E_{TiO_2} - nE_{Li}) / (n+1). \qquad (7)$$

where $E_{Li_nTiO_2}$, E_{TiO_2}, and E_{Li} are the total energies of $Li_{0.5}TiO_2$, TiO_2 and a Li atom in the structure, respectively. Based on the calculation, $E_{Li_2Ti_4O_8}$, $E_{Ti_4O_8}$, and E_{Li} were -98.078eV, -91.378eV, and -1.344 eV.

$$\Delta E = (E_{Li_2Ti_4O_8} - E_{Ti_4O_8} - 2E_{Li}) / (2+1) = 1.34\text{eV} \qquad (8)$$

Based on Supplementary Equation 8, ΔE is 1.34 eV.

Supplementary References

1. Yang Y.; Yin L.; Gong Y.; Niu P.; Wang J.; Gu L.; Chen X.; Liu G.; Wang L.; Cheng H., An Unusaual Strong Visible-Light Absorption Band in Red Antase TiO_2 Photocatalyst Induced by Atomic Hydrogen-Occupied Oxygen Vacancies, *Adv. Mater.*, **2018,** 30(6), 1704479.
2. Williamson, G.; Hall, W., X-ray line broadening from filed aluminium and wolfram. *Acta Metal.* **1953,** *1* (1), 22-31.
3. Wang, Z.; Wang, L., Progress in designing effective photoelectrodes for solar water splitting. *Chinese J. Catal.* **2018,** *39* (3), 369-378.
4. Yang, Y.; Wang, S.; Jiao, Y.; Wang, Z.; Xiao, M.; Du, A.; Li, Y.; Wang, J.; Wang, L., An unusual red carbon nitride to boost the photoelectrochemical performance of wide bandgap photoanodes. *Adv. Funct. Mater.* **2018,** *28* (47), 1805698.
5. Wang, C.; Chen, Z.; Jin, H.; Cao, C.; Li, J.; Mi, Z., Enhancing visible-light photoelectrochemical water splitting through transition-metal doped TiO_2 nanorod arrays. *J. Mater. Chem. A* **2014,** *2* (42), 17820-17827.
6. Feng, X.; Shankar, K.; Paulose, M.; Grimes, C. A., Tantalum-Doped Titanium Dioxide Nanowire Arrays for Dye-Sensitized Solar Cells with High Open-Circuit Voltage. *Angew. Chem. Int. Ed.* **2009,** *48* (43), 8095-8098.
7. Pan, L.; Zou, J.-J.; Zhang, X.; Wang, L., Photoisomerization of norbornadiene to quadricyclane using transition metal doped TiO_2. *Ind. Eng. Chem. Res.* **2010,** *49* (18), 8526-8531.
